# Expression of ABCB1, ABCC1, and LRP in Mesenchymal Stem Cells from Human Amniotic Fluid and Bone Marrow in Culture—Effects of In Vitro Osteogenic and Adipogenic Differentiation

**DOI:** 10.3390/ijms26020510

**Published:** 2025-01-09

**Authors:** Carolina Martinez Romão, Felipe de Lara Janz, Jorge Luis Maria Ruiz, Marco Antônio Borges Lopes, Alexandre Fogaça Cristante, Tarcísio Eloy Pessoa de Barros Filho, Débora Levy, Sérgio Paulo Bydlowski

**Affiliations:** 1 Lipids, Oxidation, and Cell Biology Group, Laboratory of Immunology (LIM19), Heart Institute (InCor), Hospital das Clínicas, Faculdade de Medicina, Universidade de São Paulo (HCFMUSP), São Paulo 05403-900, Brazil; krolroma@gmail.com (C.M.R.); d.levy@hc.fm.usp.br (D.L.); 2 Hospital Sírio-Libanês, São Paulo 01308-050, Brazil; 3 General Biology Department, State University of Ponta Grossa (UEPG), Ponta Grossa 84010-330, Brazil; fljanz@uepg.br; 4 Latin American Institute of Life and Natural Sciences, Federal University for Latin American Integration (UNILA), Foz do Iguaçu 85870-650, Brazil; jorge.ruiz@unila.edu.br; 5 Laboratory of Obstetric Physiology, Department of Obstetrics and Gynecology, Faculdade de Medicina FMUSP, Universidade de São Paulo, São Paulo 01246-903, Brazil; marco.lopes@hc.fm.usp.br; 6 Department of Orthopedics and Traumatology, Faculdade de Medicina FMUSP, Universidade de São Paulo, São Paulo 05402-000, Brazil; aacristante@uol.com.br (A.F.C.); tarcisio.barros@hc.fm.usp.br (T.E.P.d.B.F.); 7 National Institute of Science and Technology in Regenerative Medicine (INCT-Regenera), CNPq, Rio de Janeiro 21941-902, Brazil

**Keywords:** mesenchymal stem cell, ABC transporters, multidrug resistance, P-glycoprotein, amniotic fluid, bone marrow, cell differentiation

## Abstract

Mesenchymal stem cells (MSCs) are multipotent cells with the potential to differentiate into various lineages. They have also the potential to protect themselves against harmful stimuli to maintain their functional integrity. Drug resistance-related transporters such as ABCB1 (P-glycoprotein; P-gp), ABCC1 (MRP1; multidrug resistance-related Protein 1), and LRP (lung resistance protein) may protect MSCs against toxic substances such as chemotherapeutic agents. This study evaluated ABCB1, ABCC1, and LRP before and after the differentiation of MSCs derived from human amniotic fluid (AF) and bone marrow (BM). P-gp expression in both AFMSCs and BMMSCs was analyzed by immunocytochemistry, and pump function was analyzed by cell viability assay with doxorubicin (DOX) and Rhodamine 123 (Rh 123) dye exclusion. ABCB1, ABCC1, and LRP gene expression was determined by RT-PCR both before and after osteogenic and adipogenic differentiation. The MES-SA/DX5 cell line was used as a model of resistance to DOX and the overexpression of P-gp. Both AFMSCs and BMMSCs displayed a high P-gp expression, although lower than MES-SA/DX5 control cells. It was shown that both, undifferentiated AFMSCs and BMMSCs, have high cell viability in response to DOX, similar to the MES-SA/DX5 lineage. ABCB1 was less expressed in BM than in AFMSCs in undifferentiated samples, while no differences were observed in the expression of ABCC1 and LRP. AFMSCs showed an increase in ABCB1 after osteogenic differentiation, whereas BMMSCs exhibited lower ABCB1 and ABCC1 expression after osteogenic and adipogenic differentiation. The findings suggest that ABCB1, ABCC1, and LRP gene expression in AFMSCs and BMMSCs is influenced by differentiation processes and further support the concept that these transporters modulate MSC differentiation in a cell source-dependent way.

## 1. Introduction

Multidrug resistance (MDR) is defined as the resistance of cells to drugs. It is promoted by different protein structures that are involved in the cell efflux of a wide variety of substrates such as oligosaccharides and proteins. The mechanisms of MDR include increased xenobiotic metabolism, enhanced drug efflux (such as antineoplastic drugs), growth factors, increased DNA repair capacity, and genetic factors [1]. MDR is primarily facilitated by protein structures that mediate the efflux of diverse substrates, and the excretion of toxic compounds, thereby reducing the accumulation of harmful substances within cells [2,3].

Among these proteins, multidrug resistance transporters that are part of the ABC (adenosine triphosphate-binding cassette) superfamily are the most studied [2,4]. ABC transporters are transmembrane proteins localized in the plasma and organelle membranes that utilize the energy from ATP hydrolysis to actively export specific substrates across the cell membranes [5]. These proteins are usually overexpressed in cells that exhibit an MDR phenotype.

Humans express seven ABC subfamilies with at least 49 members [6]. Among them, the human multidrug resistance protein 1 (MDR1/P-glycoprotein/P-gp), encoded by the ABCB1 gene, and multidrug resistance-associated protein 1 (MRP1), encoded by the ABCC1 gene, increase the efflux of chemotherapeutic drugs, such as doxorubicin (DOX), leading to drug resistance in cancer patients [6,7,8,9].

Another protein is the ribonucleoprotein lung resistance-related protein (LRP), also known as the major vault protein (MVP or VAULT1), which has been described as a drug efflux transporter and is credited with contributing to chemoresistance [3,8].

In addition to being responsible for the extrusion of cytotoxic compounds, these transporters may also be involved in protective mechanisms and signal transduction pathways that regulate proliferation, differentiation, and apoptosis in several cell types, including mesenchymal stem cells (MSCs) [6]. These cells are one of the key components of the tumor microenvironment in which they can regulate transporter expression.

Mesenchymal stem cells are multipotent cells derived from various fetal and adult tissues, capable of self-renewal and differentiation in several cell lineages under appropriate conditions [10,11,12]. Therefore, they are quite important in tissue regeneration and repair.

In vitro, according to a Society for Cellular Therapy publication, MSCs are described as cells capable of adhering to plastic, with a fibroblast-like morphology, capable of self-renewal and differentiation into chondrogenic, osteogenic, and adipogenic lineages [13,14,15,16]. They also express pluripotency factors associated with embryonic stem cells, such as Oct-4 and Nanog [6]. MSCs are also characterized by the expression of specific membrane proteins such as CD105, CD73, and CD90, while lacking the expression of CD45, CD34, and CD14 [6,10,15,17].

MSCs are a crucial cellular component of the bone marrow (BM) microenvironment, supporting hematopoiesis. Their primary role is to maintain and regulate the properties of hematopoietic stem cells (HSCs). The interaction between MSCs and HSCs prevents HSC differentiation and protects them from apoptosis, thereby promoting self-renewal and maintaining stemness [18,19].

The human amnion, along with amniotic fluid (AF), protects the fetus from trauma, infections, and toxic agents. AF consists of water, electrolytes, lipids, proteins, hormones, and cells. The cells in AF include a heterogeneous population derived from the embryo, particularly from the amniotic membrane, and the respiratory, intestinal, and urinary tracts. MSCs can also be isolated from AF (AFMSCs), and these cells may have pluripotent potential similar to embryonic stem cells (ESCs) or induced pluripotent stem cells (iPS) [12,15,20,21,22,23].

During the differentiation of MSCs into specific cell types, a variety of stimuli and inhibitors play critical roles in both the initial commitment and the later stages of differentiation. In osteogenic differentiation, Runx2 is a key transcription factor that promotes osteoblast differentiation while inhibiting adipogenic and chondrogenic differentiation [14,16]. The expression of Runx2 is regulated by several signaling pathways, including Wnt, BMP, and Notch [14,16]. Adipogenic differentiation is primarily controlled by PPARγ, which works in conjunction with other transcription factors to promote the expression of adipogenic markers [14,16].

Relatively few reports examined the presence and potential functions of membrane transporter proteins in MSCs and most were related to those from bone marrow, mainly in cancer [24,25,26,27,28]. In fact, another notable property of MSCs is the expression of specific ABC transporters, which enable them to efflux certain chemotherapy drugs [2,3,11]. Bone marrow-derived MSCs (BMMSCs) have been shown to exhibit resistance to chemotherapeutic agents commonly used in bone marrow transplantation (e.g., busulfan, cyclophosphamide, and methotrexate). However, they have also shown relative sensitivity to cytotoxic agents such as paclitaxel, vincristine, etoposide, and cytarabine. The mechanisms underlying these differing responses to chemotherapeutic agents remain poorly understood [6,17,18].

Some of those studies suggested that MDR phenotypes are different in MSCs, depending on their origin, and that some related-proteins could affect self-renewal ability. In addition, the expression of certain ABC genes could be associated with the differentiation of MSCs [29,30,31].

Based on these considerations, this study aimed to evaluate the expression of specific efflux pumps, namely ABCB1, ABCC1, and LRP, in human normal bone marrow- and amniotic fluid-derived MSCs before and after adipogenic and osteogenic differentiation, thus providing further insight into the relationship between these structures and the differentiation process.

## 2. Results

### 2.1. MSC Characterization by Oct-4 and Nanog Expression, Differentiation Capacity, and Surface Markers

BMMSCs and AFMSCs exhibited a fibroblast-like morphology in culture. BMMSs and AFMSCs expressed both Oct-4 and Nanog and were shown to be able to differentiate into osteogenic and adipogenic lineages in vitro.

BMMSCs and AFMSCs exhibited typical surface markers associated with mesenchymal cells (CD105, CD29, CD44, and CD90) and showed little expression of non-mesenchymal surface markers (CD45, CD31, CD34, and CD14).

### 2.2. Characterization of MDR in AFMSCs and BMMSCs Measured by P-gp Expression, Cell Viability Under Doxorubicin Exposure, and Dye Exclusion

#### 2.2.1. P-gp Is Expressed in AF and BMMSCs as Measured by Immunocytochemistry

**Figure 1:**
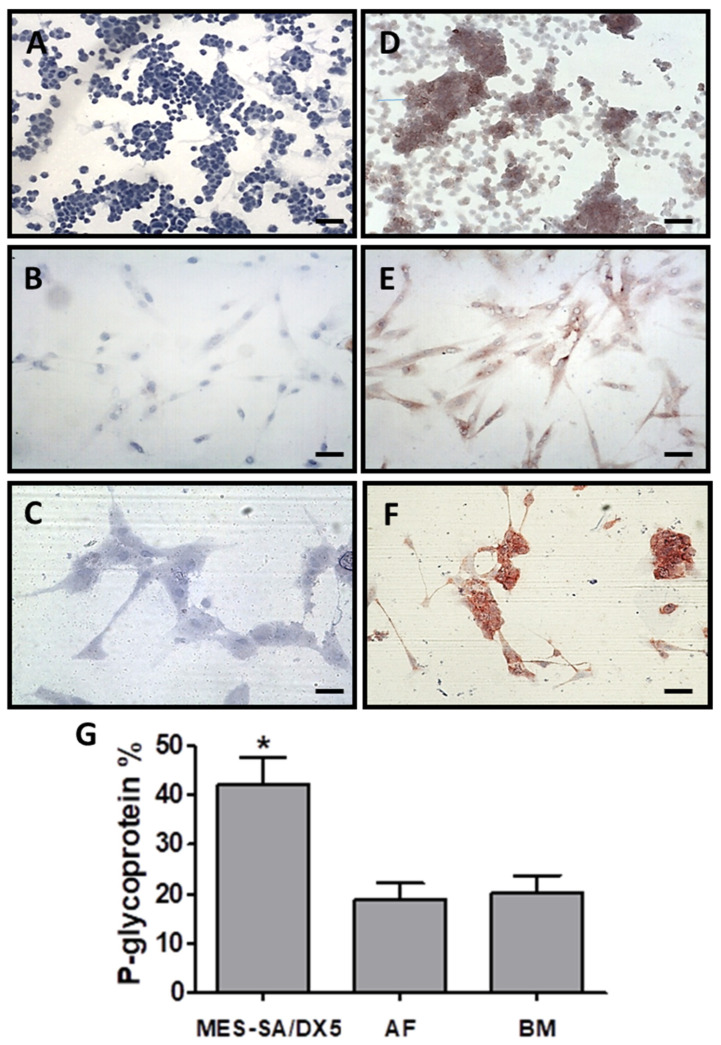
Optical microscopy visualization of immunocytochemistry staining for P-gp revealed with AEC (100×): (**A**) MES-SA/DX5 control, (**B**) AFMSC control, and (**C**) BMMSC control; P-gp immunocytochemistry (100×): (**D**) MES-SA/DX5, (**E**) AFMSCs, and (**F**) BMMSCs. All cell nuclei were counterstained with H&E. Scale bar: 50 µM. (**G**) Quantification of P-gp area positivity (%) in immunocytochemistry of undifferentiated AFMSCs (*n* = 3), BMMSCs (*n* = 3), and MES-SA/DX5 (*n* = 3). The Kruskal–Wallis test was applied, followed by Dunn’s post-test, * *p* = 0.0052.

P-gp was detected by immunocytochemistry in undifferentiated AFMSCs (Figure 1B,E) and BMMSCs (Figure 1C,F), as well as in MES-SA/Dx5 control cells (Figure 1A,D). However, the expression area (%) of P-gp in AFMSCs and BMMSCs was lower compared to the MES-SA/DX5 control cells (Figure 1G).

#### 2.2.2. Viability of AF and BMMSCs Under Treatment with Doxorubicin Is Similar to That of Control Cells Expressing P-gp

**Figure 2:**
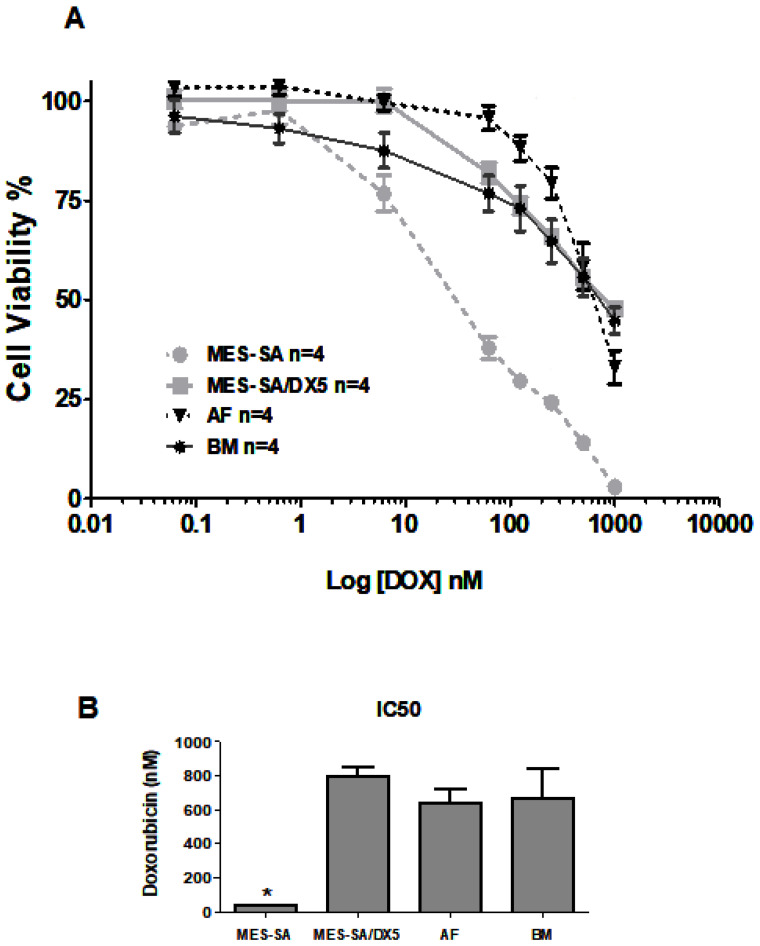
(**A**) Cell viability curve (%) of MES-SA (*n* = 4) (negative control), MES-SA/DX5 (*n* = 4) (positive control), AFMSCs (AF) (*n* = 4), and BMMSCs (BM) (*n* = 4) exposed to different concentrations of doxorubicin (0.01–10,000 nM, 6 replicates each) for 72 h; (**B**) doxorubicin (DOX) concentration (nM) was required to eliminate 50% of the cell population, calculated through the cell viability curve. The Kruskal–Wallis test was applied, followed by Dunn’s post-test, * *p* = 0.0302.

Figure 2 shows the viability of cells submitted to DOX treatment. MES-SA cells reached 100% cell death at the highest DOX concentration used in the experiment (1000 mM), while MES-SA/Dx5, AFMSCs, and BMMSCs maintained significant levels of viability (47.81%, 33.12%, and 44.83%, respectively).

#### 2.2.3. Rhodamine 123 Dye Exclusion in AF and BMMSCs Is Similar to That of Control Cells Expressing P-gp

**Figure 3:**
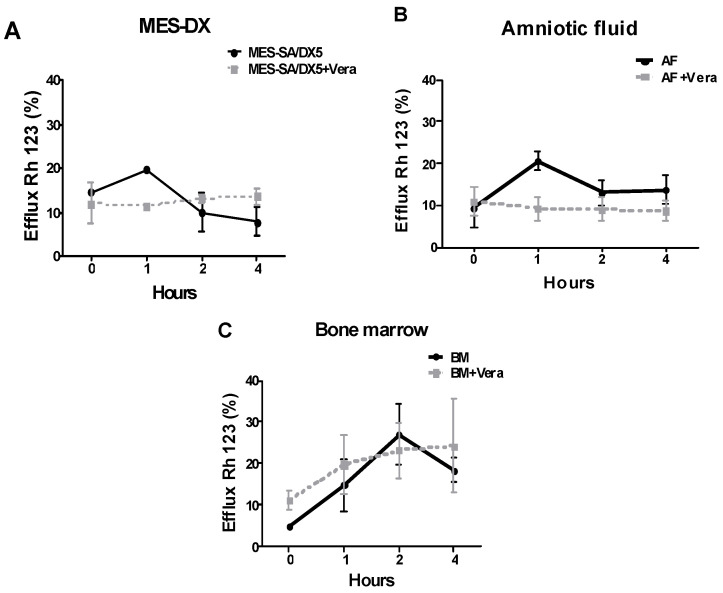
Efflux of rhodamine 123 dye (Rh 123) shown as means ± SD of the percentages (%) obtained by fluorescence flow cytometry of (**A**) MES-SA/DX5 (resistant) (*n* = 3), (**B**) amniotic fluid (AF) (*n* = 3), and (**C**) bone marrow (BM) (*n* = 3), internalized with Rh 123 (2 mM) subsequently subjected to excluding periods of 0, 1, 2, and 4 h with or without verapamil; test applied was Two-way ANOVA, post-test was Bonferroni.

Rhodamine 123 exclusion experiments showed that verapamil, after 1 h of incubation, decreased the efflux of rhodamine in amniotic fluid MSCs and MES-SA/Dx5 cells by 50%, although not significantly, while a less important effect was observed in bone marrow MSCs only after 2 h of incubation (Figure S1 and Figure 3).

### 2.3. AF and BMMSCs Express ABCB1, ABCC1, and LRP Genes: Effect of Osteogenic and Adipogenic Differentiation

**Figure 4:**
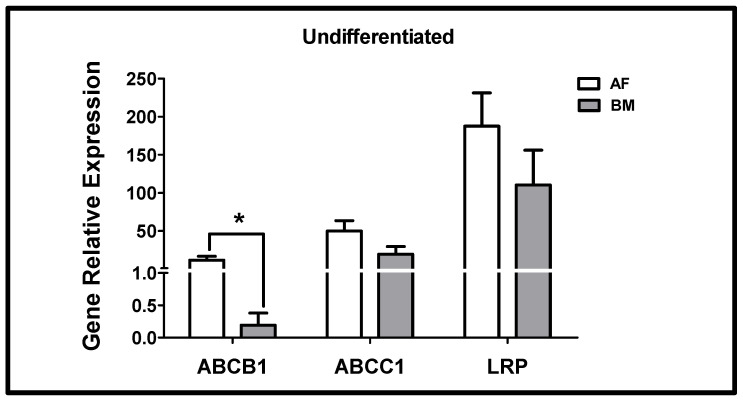
ABCB1, ABCC1, and LRP gene expression in undifferentiated AFMSCs (*n* = 9) and BMMSCs (*n* = 6); Mann–Whitney test was applied, * *p* = 0.0008.

Both undifferentiated AFMSCs and BMMSCs expressed the ABCB1, ABCC1, and LRP genes. The expression of ABCB1 was higher in AFMSCs compared to BMMSCs. However, in these cells, ABCC1 and LRP gene expression was not statistically different (Figure 4).

**Figure 5:**
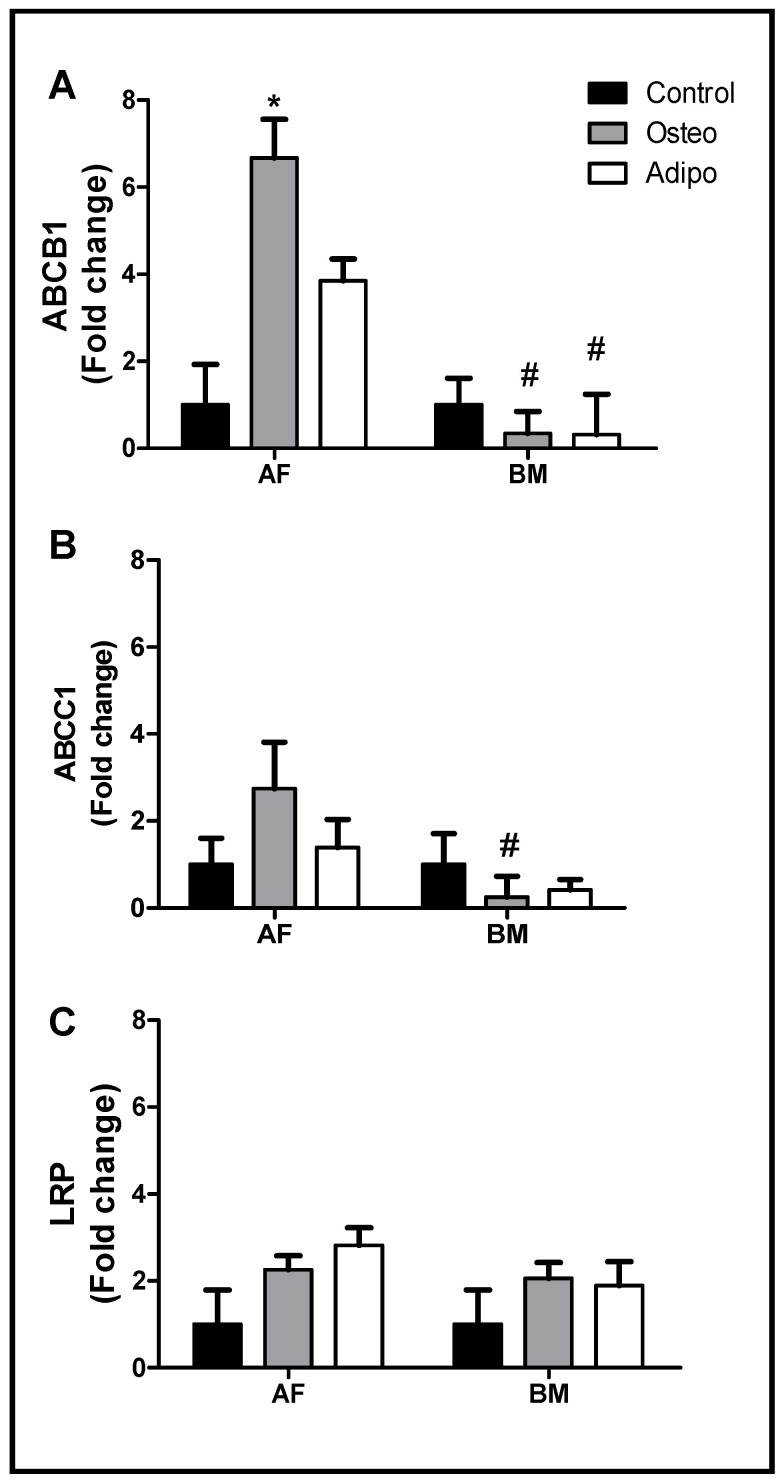
(**A**) ABCB1, (**B**) ABCC1, and (**C**) LRP gene expression in AFMSCs (AF) and BMMSCs (BM) after adipogenic (LA = 3; MO = 4) and osteogenic differentiation (LA = 4; MO = 4). Undifferentiated cells were used as controls. Two-way ANOVA was applied. * *p* = 0.001, # *p* = 0.05.

Figure 5 shows that there was a tendency for an increase in the expression of the ABCB1 and ABCC1 genes promoted by osteogenic and adipogenic differentiation in AFMSCs. However, a significant level was achieved only for ABCB1 with osteogenic differentiation. In contrast, no changes were observed in LRP expression.

In BMMSCs (Figure 5), on the contrary, there was a tendency towards a decrease in the expressions of ABCB1 and ABCC1, which were significant only with the osteogenic differentiation. Again, no changes were observed in the LRP gene expression in any situation.

## 3. Discussion

Cultured cells from bone marrow and amniotic fluid exhibited a fibroblast-like morphology and expressed both Oct-4 and Nanog. They also exhibited typical surface markers associated with mesenchymal cells (CD105, CD29, CD44, and CD90) and showed either no or weak expression of non-mesenchymal surface markers (CD45, CD31, CD34, and CD14). These findings confirm that they are of mesenchymal origin, together with their ability to differentiate into osteogenic and adipogenic lineages in vitro [13].

P-gp was detected by immunocytochemistry in undifferentiated AFMSCs and BMMSCs, as well as in MES-SA/Dx5 control cells. As expected, the expression of P-gp in AFMSCs and BMMSCs was lower compared to the MES-SA/DX5 control cells. In the cytotoxicity curve analysis, MES-SA cells reached 100% cell death at the highest DOX concentration used in the experiment (1000 mM), while MES-SA/Dx5, AFMSCs, and BMMSCs maintained significant levels of viability (47.81%, 33.12%, and 44.83%, respectively), showing the resistance to DOX. Similar findings were shown by others testing BMMSCs when these cells were exposed to various chemotherapeutic agents [32].

For the evaluation of P-gp function, Rh 123 exclusion analysis was performed. In this test, AFMSCs exhibited dynamics similar to those of MES-SA/DX5 resistant cells, with an efflux peak after 1 h and a reduction in efflux after 2 and 4 h of incubation. High dye exclusion during the first hour of incubation has also been reported by other authors in Caco-2 cells [32]. In contrast, BMMSCs behaved differently, showing progressive Rh 123 exclusion throughout all incubation periods, both with and without verapamil. Similar results have also been reported in a stromal cell line from mouse BM grown in the presence of doxorubicin, showing the ability of these cells to internalize a large amount of the drug without significant toxicity [33]; when co-cultured, these DOX-saturated cells inhibited the growth of other cell types, acting as a chemotherapy reservoir and subsequently releasing the drug or its metabolites. These findings suggest that P-gp may not be the only factor contributing to MSCs’ resistance to DOX. Human BMMSCs may possess additional mechanisms against toxic agents beyond P-gp. These mechanisms could include the reduction or blockage of toxic compounds or the ability to adsorb and release them later. Therefore, our data suggest that the protective mechanisms of MSCs may differ between the two sources analyzed in this study. For instance, the gene signatures for ABC transporters were found to be radically different between hematopoietic stem cells (HSCs) and other types of stem cells, thereby suggesting that HSCs rely on a different repertoire of these transporters as compared to other tissue/cell types [34].

Both undifferentiated AFMSCs and BMMSCs expressed the ABCB1, ABCC1, and LRP genes. Gene expression was higher in AFMSCs compared to BMMSCs; however, only the expression of the ABCB1 gene was statistically significantly higher in AFMSCs than in BMMSCs. This finding conflicts with immunocytochemistry results, which show no difference in P-gp expression between AFMSCs and BMMSCs. This discrepancy may suggest that regulatory mechanisms involving pre-existing mRNA could affect P-gp distribution.

We observed that the osteogenic and adipogenic differentiation of BMMSCs and AFMSCs influenced the expression of the ABCB1 and ABCC1 genes. In AFMSCs, the expression of all genes increased with both differentiation processes, although statistical significance was only reached for ABCB1 expression in osteogenic differentiated cells. In contrast, BMMSCs showed a lower expression of the ABCB1 and ABCC1 genes after osteogenic and adipogenic differentiation. LRP gene expression was not affected by osteogenic and adipogenic differentiation of both BMMSCs and AFMSCs.

Similar to our findings, others have suggested that ABCB1 gene expression may decrease during the differentiation process in BMMSCs [29]. In another study, the authors speculated that ABCB1 gene downregulation occurs during chondrogenic differentiation of MSCs, alongside an increase in type II collagen expression [31].

These regulatory mechanisms may be related to specific functions that MSCs perform in their tissue of origin or their associated in vitro behavior, which can vary between cell lines. For example, AFMSCs exhibit higher replicative rates and differentiation potential compared to adult MSCs in culture, along with the high expression of undifferentiation genes and elevated telomerase activity, similar to embryonic stem cells [35]. As a result, the high activity of these cells in vitro may require greater energy demand, potentially mediated by P-gp activity in transporting ions, nutrients, and hydrophobic compounds like cholesterol. Understanding the mechanisms that regulate P-gp expression in MSCs is crucial for gaining insight into the physiological proteomic modulation of MSCs and enhancing our knowledge of drug resistance.

Despite high levels of LRP gene expression in both AFMSCs and BMMSCs, followed by ABCC1, the ABCB1 gene showed the lower expression in both MSC sources. It is suggested that other proteins may have been underestimated in drug resistance compared to P-gp. Given these assumptions, MRP1 and LRP may play a protective role in MSCs [36,37].

It is notable that LRP gene expression is high in MSCs, but its exact function in these cells has not yet been fully defined. However, some authors suggest that this pump may play a role in cell plasticity. For example, it has been shown that blocking LRP expression in dendritic cells led to decreased cell viability and issues with the maturation of young cells [38]. Additionally, LRP was linked to embryonic development by studying mouse embryo fibroblasts lacking LRP [38]. These fibroblasts, when cultured under optimal conditions, showed no proliferative activity, and, in the absence of fetal serum, exhibited increased apoptosis. These findings suggest a relationship between LRP expression and self-renewal ability.

Therefore, stem cells have been recognized as an important tool in the treatment of several diseases, including cancer. Recently, AFMSCs have been evaluated as having superior multipotency and immunomodulatory properties compared to MSCs derived from other tissues, such as bone marrow [39,40,41,42,43]. Furthermore, in diseases such as cancer, the expression of multidrug resistance in stem cells may be important [44,45]. Here, we have evaluated some of the characteristics of multidrug resistance in stem cells from amniotic fluid and bone marrow.

## 4. Materials and Methods

### 4.1. Isolation and Culture of MSCs

This study was conducted in accordance with the Declaration of Helsinki, and the protocol was approved by the Ethics Committee of the Institution (Hospital das Clínicas, Faculdade de Medicina, University of São Paulo, Brazil), CAPPesq, numbers 620/11 and 0410/09, and HCFMUSP 8/41. Written informed consent was obtained from all subjects before they participated in this study.

MSCs from amniotic fluid (AF) and bone marrow (BM) were obtained as described [12,15,18,21,22]. Briefly, amniotic fluid was collected from nine healthy patients at 15 to 18 weeks of gestation via amniocentesis for fetal karyotype diagnosis at the Obstetrics Service of the same institution. AF samples (2–5 mL) were centrifuged at 450× *g* for 10 min at room temperature, and the resulting cells were washed with PBS. Cells were cultured in minimal essential medium-α modification (α-MEM) (Sigma-Aldrich, St. Louis, MO, USA) supplemented with 20% heat-inactivated FBS (Vitrocell, Waldkirch, Germany) and 1% penicillin/streptomycin antibiotics (100 UI/mL and 100 µg/mL, respectively; Sigma Aldrich, Saint Louis, MO, USA). The cells were maintained in a 37 °C incubator with a humidified atmosphere of 5% CO_2_, with the culture medium changed every 3–4 days for approximately 12 days, when the first colonies appeared. After the 4th to 6th passages, cells were frozen under liquid nitrogen.

Bone marrow samples were obtained via iliac crest aspiration from six healthy donors undergoing bone surgery in the Orthopedics Department. BM samples (2–10 mL) were diluted 1:2 (*v*:*v*) in PBS and centrifuged for 7 min at 700× *g* at room temperature and washed with PBS. Mononuclear cells were isolated using Ficoll-Paque PLUS (GE Healthcare Life Sciences, Chicago, IL, USA), washed, plated in 75-cm^2^ culture flasks (Santa Cruz Biotechnology, Dallas, TX, USA), and cultivated in an MSC medium. The MSC medium consisted of low-glucose Dulbecco’s Modified Eagle Medium (DMEM) supplemented with 20% heat-inactivated FBS and 1% penicillin/streptomycin antibiotics, as described above. After transferring to flasks, the cells were incubated at 37 °C in a humidified atmosphere of 5% CO_2_, with the culture medium changed every 3–4 days. Before reaching confluence, cells were detached using a trypsin-EDTA solution (Gibco, Waltham, MA, USA) and seeded at a density of 5 × 10^3^ cells/cm^2^. After the 4th to 6th passages, cells were frozen under liquid nitrogen.

BMMSCs and AFMSCs were kept frozen under liquid nitrogen until use. After thawing, cells from the second and third passages were used for the experiments.

### 4.2. MSC Characterization and Differentiation

AFMSCs and BMMSCs were characterized through culture surface adherence properties and showed a fibroblast-like morphology. The full characterization comprised the expression of undifferentiated marker genes (Oct-4 and Nanog) and surface antigen analysis.

#### 4.2.1. Expression of Oct-4 and Nanog by RT-PCR

Total mRNA was extracted from AFMSCs and BMMSCs using Trizol (Invitrogen, Gaithersburg, MD, USA) following the manufacturer’s instructions. The purity of the RNA was assessed by determining the absorbance ratio at 260 nm and 280 nm. Samples were treated with the RQ1 RNase-Free DNase kit (Promega, Madison, WI, USA) to eliminate genomic DNA. A one-step reaction was then performed using the SuperScript III Platinum One-Step RT-PCR kit (Invitrogen, Gaithersburg, MD, USA). This process included an initial reverse transcription step at 50 °C for 30 min, followed by denaturation at 95 °C for 5 min in a gradient thermal cycler (PTC-200, MJ Research, Watertown, NY, USA). The reaction then underwent 35 cycles consisting of denaturation at 94 °C for 30 s, annealing at 57 °C for 30 s, and extension at 72 °C for 1 min, with a final extension step at 72 °C for 8 min. The sense and antisense primer sequences used in this study were as follows: Oct-4: 5′-AAG CGA TCA AGC AGC GAC TAT-3′ and 5′-GGA AAG GGA CCG AGG AGT ACA-3′; Nanog: 5′-CAA AGG CAA ACA ACC CAC TT-3′ and 5′-TCT GCT GGA GGC TGA GGT AT-3′. Total RNA from the Ntera-2.c1D1 (human pluripotent teratocarcinoma cell line) was used as a positive control for the undifferentiated state of MSCs. The human β-actin gene served as an endogenous control in all reactions. RT-PCR products were analyzed on a 2% agarose gel stained with ethidium bromide and visualized under ultraviolet light.

#### 4.2.2. Evaluation of MSC Surface Markers by Immunophenotyping

The surface membrane proteins of AFMSCs and BMMSCs were evaluated using flow cytometry (FacsCalibur, Becton and Dickinson, San Jose, CA, USA) [12,21]. After trypsinization and washing with PBS, approximately 5 × 10^5^ cells were stained for 30 min in a dark room with primary monoclonal antibodies (Invitrogen, USA) labeled with fluorochromes: fluorescein isothiocyanate (FITC)—CD34 (clone 581), CD44 (clone MEM-85), CD45 (clone HI30), and CD90 (clone 30-H12); or phycoerythrin (PE)—CD14 (clone TuK4), CD29 (clone MEM-101A), CD31 (clone MBC78.2), CD105 (clone MJ7/18). IgG1-FITC and IgG1-PE antibodies were used as isotype controls. MSCs were fixed in 1% paraformaldehyde. Ten thousand events per sample were acquired using a FACS Calibur (Becton Dickinson, San Jose, CA, USA), and the data were analyzed using CellQuest Pro software version 3.3 (Becton Dickinson, San Jose, CA, USA).

#### 4.2.3. MSC Differentiation Assays

AFMSCs and BMMSCs were characterized for their osteogenic and adipogenic differentiation capabilities in vitro. Osteogenic differentiation was carried out using an α-MEM medium supplemented with 20% FBS, 100 nM of dexamethasone, 50 mM of ascorbic acid, and 10 mM of β-glycerophosphate (all from Sigma-Aldrich, Darmstadt, Germany) for 3 weeks. Adipogenic differentiation was induced using an α-MEM medium containing 20% FBS, 10^−2^ μM of dexamethasone, 50 mM of isobutylmethylxanthine, 200 mM of indomethacin, and 0.5 mg/mL of insulin (Sigma-Aldrich) for 3 weeks. The medium was changed twice a week. After 21 days, differentiation was confirmed by staining with Oil Red O for adipogenic differentiation and Alizarin Red for osteogenic differentiation.

### 4.3. MSC Multidrug Resistance Characterization

#### 4.3.1. P-gp Determination in MSCs by Immunocytochemistry

MSCs were cultured at a density of 1 × 10^4^ cells on cell culture chamber slides (MSC, Dublin, Ireland) for 3 days. The cells were then fixed in cold acetone for 7 min and air-dried at room temperature. The slides were stored at −20 °C until the assay was performed. For immunocytochemistry, the Novo Link Polymer Detection System kit (Novocastra, Newcastle, UK) and the mouse anti-human P-glycoprotein surface epitope antibody (JSB-1 clone; Zymed, San Francisco, CA, USA) were used. Following the antibody reaction, the slides were stained with the chromogen substrate AEC (3-amino carbazole ethyl; Sigma-Aldrich, Saint Louis, MO, USA) for 30 min and counterstained with H&E. Image analysis was performed using light microscopy and the Image-Pro Plus 7.0 software (PerkinElmer, Waltham, MA, USA). Twenty-five fields were analyzed per slide, and P-gp expression was quantified as the percentage of the stained area.

MES-SA (a cell line derived from human uterine sarcoma, susceptible to doxorubicin due to P-gp downregulation) and MES-SA/Dx5 cells (a multidrug-resistant cell line derived from human uterine sarcoma, resistant to doxorubicin due to P-gp upregulation) were obtained from the American Type Culture Collection (ATCC CRL-1976 and ATCC-CRL-1977) and used as negative and positive controls for P-gp expression, respectively. These cells were maintained in McCoy’s 5A medium (Sigma-Aldrich, Saint Louis, MO, USA) supplemented with 10% heat-inactivated FBS and antibiotics.

#### 4.3.2. MSC Viability Assay Response to Doxorubicin

To evaluate the resistance of MSCs to doxorubicin treatment, which serves as a measure of P-gp function, the MTT assay (Sigma-Aldrich, Saint Louis, MO, USA) was performed following the method described by Carmichael [46]. AFMSCs, BMMSCs, MES-SA, and MES-SA/Dx5 cells were plated in 96-well plates at a density of 1 × 10^3^ cells per well and treated with doxorubicin at final concentrations up to 1000 nM. The amount of formazan produced was measured at 570 nm with a reference wavelength of 620 nm using an ELX 800 Microplate Reader (Biotek, Winooski, VT, USA). The experiment was repeated four times, with six replicates for each doxorubicin concentration. The IC50 values were determined by fitting the data to a variable slope curve using GraphPad Prism software version 5.0 (La Jolla, CA, USA).

#### 4.3.3. Rhodamine 123 Dye Exclusion Assay

P-gp function was assessed by measuring the exclusion of Rhodamine 123 dye (Rh 123, Sigma-Aldrich, Saint Louis, MO, USA). AFMSCs, BMMSCs, and MES-SA/Dx5 cells were cultured in an α-MEM medium with 20% FCS and 2 mM of Rhodamine 123 for 1 h. After washing with PBS containing 2 mM of Rh 123, the cells were centrifuged and resuspended in PBS at a density of 1 × 10^5^ cells per tube, with or without the addition of verapamil (200 μM, Sigma-Aldrich, Saint Louis, MO, USA). The tubes were then incubated on ice for 10 min and at 37 °C for 1, 2, and 4 h. Cell fluorescence was quantified by flow cytometry using a FACSCalibur with CellQuest Pro software version 3.3. The analysis was based on the dynamics of the low fluorescence population.

### 4.4. ABCB1, ABCC1, and LRP Gene Expression in MSCs: Effect of Differentiation

AFMSCs and BMMSCs were used immediately before and after the differentiation protocols. At the end of the experimental periods, total RNA was extracted from the cells using Trizol. The samples were then treated with the RQ1 RNase-Free DNase kit. Real-time PCR was performed on a Rotor Gene 3000 instrument (Corbett Research, USA) using the SuperScript III Platinum SYBR Green One-Step qRT-PCR kit. The primer sequences used were as follows: ABCB1 forward: 5′-CCCATCATTGCAATAGCAGG-3′, ABCB1 reverse: 5′-GTTCAAACTTCTGCTCCTGA-3′; ABCC1 forward: 5′-CTGAAACCATCCATGACCTCAATCC-3′, ABCC1 reverse: 5′-GCCTCCTCGTTCACGTCCACCTGGG-3′; and LRP forward: 5′-GGGTTGTGCCCATCACCACC-3′, LRP reverse: 5′-GGTCCGCGGATGAGCCAGTGG-3′. The thermal cycler conditions included an initial incubation at 50 °C for 3 min, followed by incubation at 95 °C for 5 min. This was followed by 40 cycles of 95 °C for 15 s and 60 °C for 30 s. The products were then subjected to a melting curve analysis by heating from 60 °C to 95 °C over 20 min. To enable the relative quantification of transcripts, standard curves were generated with serial dilutions of MES-SA/Dx5 RNA. The GUSB gene was used as the housekeeping gene, and the primer sequences were as follows: forward: 5′-GAA AAT ACG TGG TTG GAG AGC TCA TT-3′ and reverse: 5′-CCG AGT GAA GAT CCC CTT TTT A-3′ [20]. The relative expression levels of ABCB1, ABCC1, and LRP transcripts were calculated using the 2^−ΔΔCT^ method [47].

### 4.5. Statistical Analysis

Values are presented as mean ± SD. Each value represents the mean of at least three independent experiments performed in triplicate. Student’s *t*-test and One-Way ANOVA were performed using GraphPad Prism software version 5.0 (GraphPad Software, La Jolla, CA, USA). The *p* values ≤ 0.05 were considered statistically significant.

## 5. Conclusions

The current amount of information regarding the expression and role of drug transporters in human stem cells is relatively limited. Here, we have shown that undifferentiated BMMSCs and AFMSCs express ABCB1, ABCC1, and LRP. Moreover, the expression of ABCB1 and ABCC1 genes is influenced by osteogenic and adipogenic differentiation.

Obviously, the present study has several weaknesses. From a theoretical point of view, the importance of the findings in relation to their physiological or pathophysiological role has not been established, as well as, for example, whether they vary in AFMSCs at different gestational periods. Or, from a methodological point of view, the study of ABCC1 and LRP did not include measurements of their more specific function or a cell line as a control.

However, the importance of the source of origin of the MSC and culturing conditions, such as early differentiation, was shown. Therefore, the regulation of stem cell biology by transporters has emerged as an important field of investigation. This knowledge could contribute for developing new approaches that could lead to improved therapeutic outcomes.

## Data Availability

The data generated in this research can be made available upon consultation with S.P.B. The data will be made available anonymously. Data from medical records of study participants cannot be made available.

## Supplementary Materials

The following supporting information can be downloaded at: https://www.mdpi.com/article/10.3390/ijms26020510/s1.
