# Supplementary material for: Expression of ABCB1, ABCC1, and LRP in Mesenchymal Stem Cells from Human Amniotic Fluid and Bone Marrow in Culture—Effects of In Vitro Osteogenic and Adipogenic Differentiation
Source: Int J Mol Sci. 2025 Jan 9;26(2):510. doi: 10.3390/ijms26020510 (PMC11765172; doi:10.3390/ijms26020510)
Supplement: Supplementary file 1 [file ijms-26-00510-s001.zip › ijms-3369850-supplementary.pdf]

Supplementary Figure S1

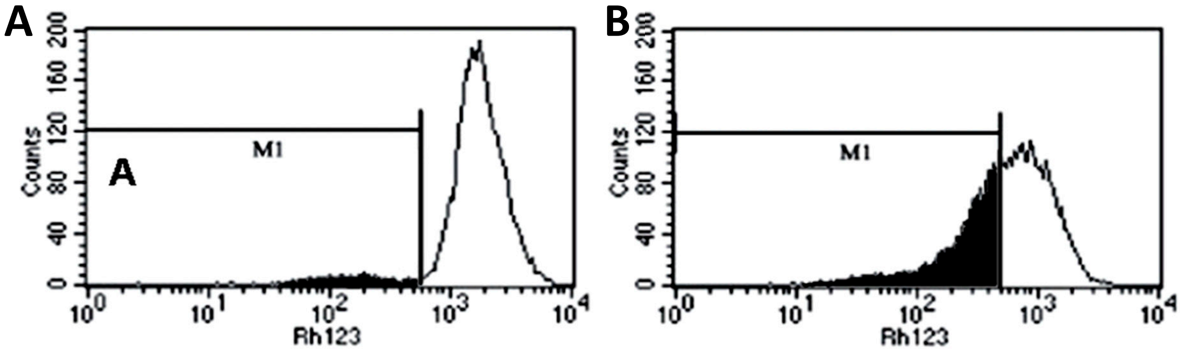

Illustration exemplifying the Rhodamine 123 (Rh123) exclusion analysis from data obtained by flow cytometry. A) Histogram of the sample subjected to exclusion at 0h, when the base marker (M1) was made; B) the same sample subjected to the exclusion period, where the required segment of the histogram shows cells that promoted dye efflux.
